# Monitoring Patient Improvement Parameters following Pasireotide Treatment in Cushing's Disease

**DOI:** 10.1155/2013/735489

**Published:** 2013-10-31

**Authors:** Chris Yedinak, Jessica Brzana, Maria Fleseriu

**Affiliations:** ^1^Northwest Pituitary Center, 3303 SW Bond Avenue, Portland, OR 97239, USA; ^2^Department of Neurosurgery, Oregon Health & Science University, 3181 SW Sam Jackson Park Road, Portland, OR 97239, USA; ^3^Department of Internal Medicine, Oregon Health & Science University, 3181 SW Sam Jackson Park Road, Portland, OR 97239, USA

## Abstract

Cushing's disease (CD) is a disorder in which chronic excess adrenocorticotropic hormone production is associated with multiple comorbidities and diminished quality of life. Postsurgical monitoring is important, and newer therapies are available for the management of surgical failure or disease recurrence. In this clinical case, we illustrate the importance of the nursing role in long-term management of CD, particularly as nurses may be the first point of contact for patients with CD. Alertness to disease signs and symptoms is crucial for timely diagnosis and improved outcomes. Successful therapy for CD requires careful monitoring of hormonal control, metabolic parameters, and therapy complications. Ongoing management requires lifelong monitoring of metabolic parameters, of side effects of treatment, and of signs of disease recurrence. Appropriate referrals may be required to facilitate overall outcomes and patient wellbeing. This patient was enrolled in a Phase III trial that was registered in the USA with clinicaltrial.gov.

## 1. Introduction

Cushing's disease (CD) is a disorder of chronic hypercortisolism secondary to excess adrenocorticotropic hormone (ACTH) production by a pituitary corticotroph adenoma [1]. It is associated with multiple comorbidities, impairment of functional status, and decreased quality of life [2, 3]. Transsphenoidal surgery is generally accepted as first-line treatment but success rates vary and relapses occur [4]. Persistent hypercortisolism may increase mortality rates compared with the general population [5, 6], which mandates long-term postsurgical biochemical monitoring.

Therapy aims at normalization of biochemical markers and the reversal of clinical features. Patients who relapse require second-line treatments including repeat surgery, radiation therapy, bilateral adrenalectomy, and/or medical therapy [7, 8]. There are several medical therapies now available. Prior to the introduction of these therapies, ketoconazole was commonly used, but it is not FDA approved for use in patients with CD, and it does not target the corticotroph tumor [8, 9]. In addition, ketoconazole could incur side effects at the high doses required to block the synthesis of cortisol. Mifepristone was recently FDA approved for use in patients with CD and concomitant hyperglycemia or diabetes mellitus. Mifepristone blocks the glucocorticoid receptor and improves glucose control, symptoms of Cushing's, and quality of life [10]. Mifepristone does not inhibit ACTH release by the underlying adenoma; rather, it competitively binds to both glucocorticoid and progesterone receptor sites. Hence, patients receiving mifepristone are monitored for therapeutic efficacy and adrenal insufficiency based on clinical signs and symptoms, as cortisol measurements during mifepristone therapy are not reliable. Mifepristone usage can result in endometrial thickening in women, or in hypertension and hypokalemia secondary to high circulating levels of cortisol [10].

The recently FDA-approved somatostatin analogue pasireotide (Signifor; Novartis, Basel, Switzerland) acts on the corticotroph tumor and decreases both ACTH and cortisol secretion. A large (*n* = 162), prospective, randomized, Phase III trial (NCT00434148) of patients with CD showed that subcutaneous (sc) pasireotide 600 and 900 *μ*g twice daily (bid) caused rapid and sustained reductions in urinary free cortisol (UFC) in the majority of patients, along with significant improvements in clinical signs and symptoms [11]. Adverse events (AEs) were similar to other somatostatin analogues with the exception of the frequency and severity of hyperglycemia. Here, we present the long-term experience of one patient with recurrent CD who was enrolled in that Phase III study.

## 2. Case Presentation

### 2.1. Initial Diagnosis

A 24-year-old Caucasian male patient presented with signs and symptoms of CD in 2004. The patient was obese (weight, 149.2 kg; body mass index, 45.47) with refractory hypertension (160/85 mmHg). His type 2 diabetes mellitus was being treated at presentation with glipizide; however, his fasting glucose at that time was 526 mg/dL.

The patient had a history of osteoporosis with several nontraumatic vertebral compression fractures. Additional history included acid reflux, hiatal hernia, migraine headaches, depression, and anxiety. This formerly athletic teen of 100 kg had gained 68 kg body weight over 3 years, despite weight-lifting and several failed diets. He reported low back pain and had suffered a spontaneous compression fracture of his lumbar spine from sneezing the previous year.

The patient reported difficulty sleeping, periodic nausea and abdominal pain, dizziness with standing, blurred vision, lower extremity cramping, alternating constipation and diarrhea, temperature dysregulation, fatigue, poor memory, feelings of social isolation, low libido, and increased thirst. His family history was negative for pituitary tumors but remarkable for hypertension, thyroid disease, diabetes type 2, alcoholism, and cardiovascular disease. He was married with a 7-month-old son. While he had a history of smoking for several years, the patient had quit prior to presentation.

On examination, the patient demonstrated a Cushingoid habitus with central weight and relative extremity sparing, marked facial rounding and plethora, dorsocervical hump, and supraclavicular fat pad filling. Significant truncal obesity with distension and gynecomastia were present along with multiple 1–1.5 cm violaceous abdominal and bilateral flank striae. Moderate gynecomastia occurred with some tenderness on palpation. Skin examination was significant for scattered ecchymosis, some skin thinning, and numerous skin tags. Proximal muscle weakness was found along with 2+ pretibial and ankle edema. Medications at that time included lisinopril, furosemide, atenolol, potassium chloride (Klor-Con), escitalopram oxalate (Lexapro), and glipizide. 

**Figure 1:**
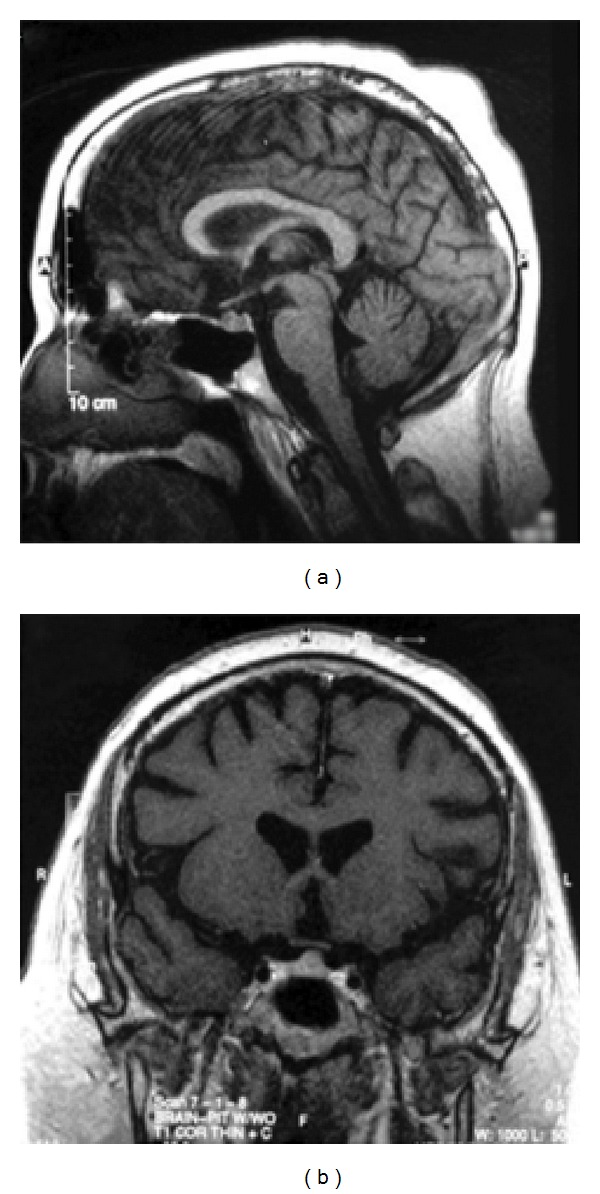
Preoperative MRI.

At presentation, the patient's mean UFC level was 262 *μ*g/24 hr (reference range < 45 *μ*g/24 hr). The patient had inappropriately low levels of follicle-stimulating hormone (1.0 mIU/mL), luteinizing hormone (1.5 mIU/L), with a low testosterone level (38 ng/dL). Prolactin (15 ng/mL), insulin-like growth factor-1 (271 ng/mL), and thyroid function were normal. A cavernous sinus sampling of ACTH levels after corticotrophin-releasing hormone (CRH) stimulation was elevated to 3650 pg/mL on the right side of the pituitary at 3 minutes and elevated to 12,368 pg/mL on the left at 5 minutes poststimulation. (A ratio central to periphery > 3 is suggestive of a pituitary source of ACTH.) Magnetic resonance imaging (MRI; Figure 1) demonstrated a 7-8 mm tumor in the superior aspect of the pituitary anterior to the infundibulum, located slightly left of midline. Optic chiasma and optic tracts were normal. 

### 2.2. Surgical Therapy

The patient subsequently underwent transsphenoidal resection using a transseptal approach to the sphenoid sinus and a left-sided hemihypophysectomy. Postsurgical midnight and morning serum cortisol levels remained elevated at 21 and 22 *μ*g/dL, respectively, as did ACTH levels of 60 pg/mL, suggesting persistent disease. 

He underwent a second surgery 3 months later, with immediate postoperative midnight and morning serum cortisol levels of 3 and 2 *μ*g/dL, consistent with disease remission. Postoperative MRI was void of residual tumor.

Over the next 12 months, the patient lost 68 kg and reported the resolution of the majority of his symptoms: abdominal striae faded, headaches resolved, and blood sugar and blood pressure normalized. HbA_1c_ was 4.8% at 3 months after surgery. Both antiglycemic and antihypertensive treatments were discontinued. The patient experienced postsurgical acute renal failure believed to be caused by adrenal insufficiency. This resolved with additional glucocorticoid replacement, and he remained glucocorticoid dependent for 8 months post operatively.

**Figure 2:**
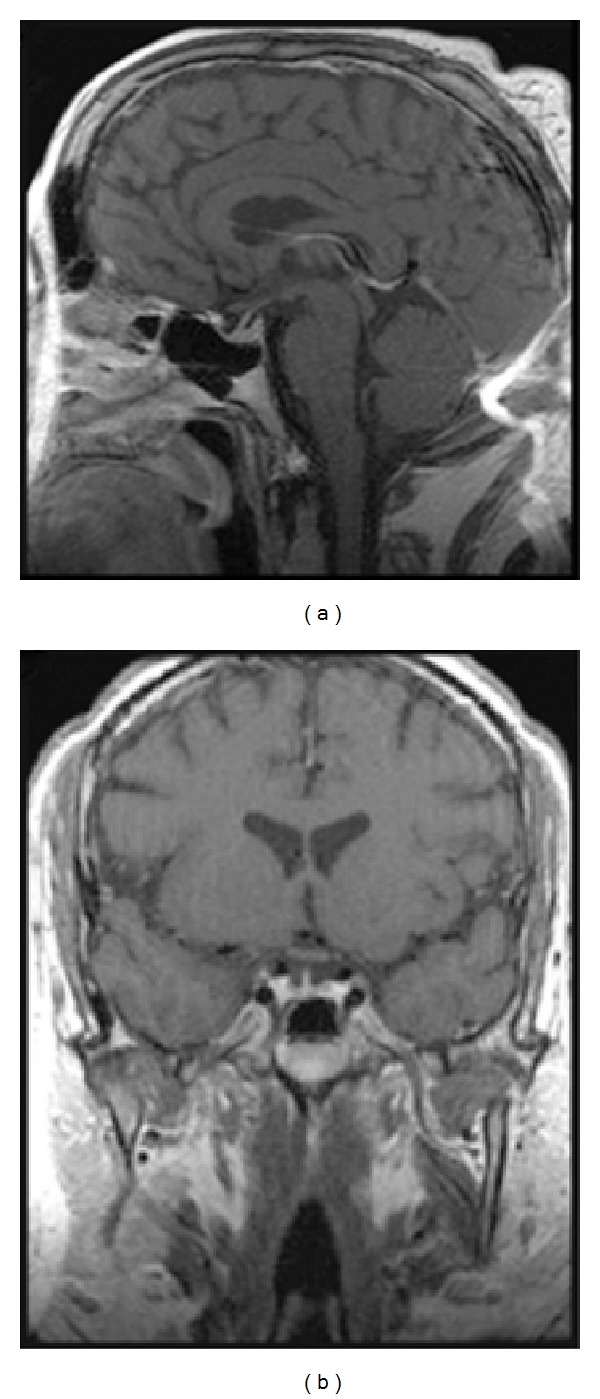
Postoperative MRI at 2 years follow-up.

After his hypothalamic-pituitary-adrenal axis recovery, midnight salivary cortisol levels, UFC levels, and ACTH levels remained normal for more than a year after the second surgery, but after 2 years, UFC was 73.6 *μ*g/24 hr. A 27-month postsurgical MRI showed a 2 mm lesion to the right of midline (Figure 2). Over the next year, the patient regained 23 kg of body weight, with a return of hyperglycemia, hypertension, anxiety, and depression. He was reluctant to pursue bilateral adrenalectomy, but there were no medical therapies approved at this time for the treatment of CD. 

### 2.3. Medical Therapy: Pasireotide

The patient consented to enrolment and treatment in a prospective, 12-month, multicenter, randomized, Phase III study (NCT00434148), which sought to assess the efficacy of pasireotide sc in patients with CD [11]. The initial screening visit confirmed elevated UFC (16.8 *μ*g/24 hr) and a positive dexamethasone/CRH test result (serum cortisol, 24.3 *μ*g/dL at 15 min; morning ACTH, 115 pg/mL). The patient was randomized to receive pasireotide 600 *μ*g bid starting in December 2007.

**Figure 3:**
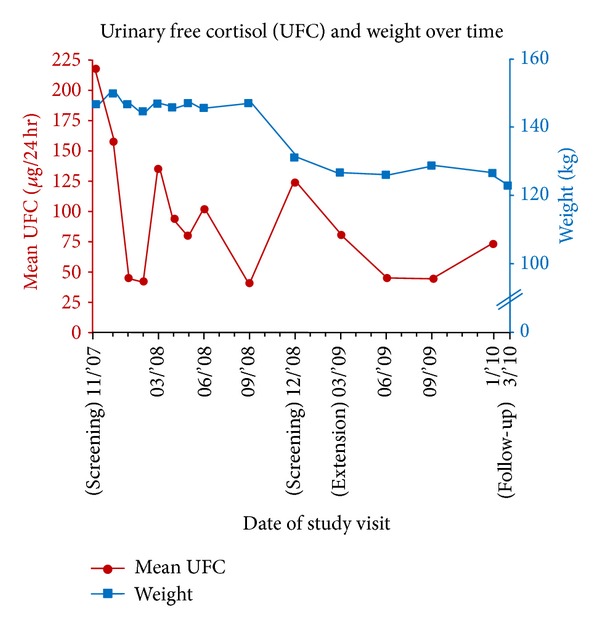
UFC and weight over time.

The patient showed a rapid response to pasireotide treatment.  Figure 3 shows mean UFC values over each 24-hour time period during the 29 months for which the patient was treated with pasireotide. UFC was dramatically reduced below the upper limit of normal (ULN) at months 1 and 2. At month 3, mean UFC level was greater than ULN (123 *μ*g/24 h), and protocol dictated that his pasireotide dosage be increased to 900 *μ*g bid.

Biochemical tests normalized during the core study (9-month UFC, 31 *μ*g/24 h; morning ACTH, 33 ng/L; serum cortisol, 19 *μ*g/dL), after which the patient lost 17 kg of body weight (Figure 3). This loss was maintained during the extension trial. The patient reported improvements in sleep, mood, and activity tolerance. He did have a brief spike in UFC levels between the core and extension studies that possibly resulted from a brief interruption of therapy secondary to drug delivery issues (owing to patient's remote location) or conversely a cyclic increase in his cortisol levels. 

During pasireotide treatments, blood pressure medications were discontinued, although lisinopril was restarted after a few months. Blood pressure remained manageable throughout the rest of the study. Bone mineral density increased numerically for both lumbar (+6.0%) and hip (+1.3%) measurements after 1 year of therapy. The Cushing's Quality of Life (QoL) questionnaire showed that pasireotide was associated with improvements in memory and healing, and with decreased irritability and bruising. Improvements in biochemical measures and clinical signs and symptoms of CD were progressive during the 2 years of pasireotide therapy.

The patient reported mild AEs related to study drug, including mild pruritus and erythema at the injection site (resolved by study week 2), nausea (resolved when patient ate immediately postinjection), and transient fasting hyperglycemia (>300 mg/dL). All AEs were managed without pasireotide dosage adjustment.

**Figure 4:**
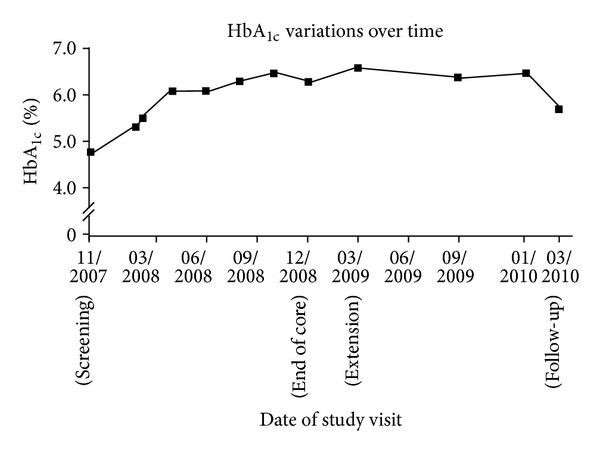
HbA_1c_ variations over time.

To manage the patient's hyperglycemia, metformin was prescribed for 2 weeks; however, this was discontinued on day 11 after it had been associated with two episodes of symptomatic hypoglycemia (40 mg/dL). The patient's HbA_1c_ level persisted below 7.0% (Figure 4). At the end of the core study, the patient's HbA_1c_ was 6.3%. Six weeks after discontinuing the study drug, the patient's HbA_1c_ normalized at 5.8%. 

As part of the clinical protocol, patients received quarterly MRI scans. During the core study, asymptomatic gallstones developed. These may occur in conjunction with the disease or with the therapy. After the patient withdrew from the study, he developed symptomatic cholecystitis and required cholecystectomy. Because the patient wanted to pursue fertility options (a study protocol exclusion), he discontinued pasireotide after more than 2 years of therapy.

## 3. Discussion

Removal of a pituitary adenoma by transsphenoidal surgery is generally accepted as the first-line treatment for patients with CD. However, the 5- and 10-year recurrence rates for CD after successful surgical resection are reportedly as high as 25% [4] and 56% [12], respectively. Based on the findings from a large, multicenter, and randomized Phase III study [11], pasireotide has recently been approved in both the US and Europe for use in adult patients with CD for whom surgery is not an option or for whom surgery has failed [13].

Pasireotide 600 and 900 *μ*g sc bid demonstrated a rapid and sustained reduction in UFC (~50% reduction in median UFC in both groups by month 2) in patients with *de novo* (nonsurgical candidates), persistent, or recurrent CD. Reductions in UFC were associated with significant improvements in clinical signs and symptoms of CD and health-related QoL [11].

In this case study, a patient with a postsurgical recurrence of CD was enrolled in the Phase III study and randomized to receive pasireotide 600 *μ*g sc bid. A rapid decrease in UFC level was observed following initiation of treatment with the patient achieving biochemical control (UFC ≤ ULN) at months 1 and 2; however, UFC rose > ULN following 3 months of treatment, leading to uptitration of the pasireotide dose. Despite significant variation, UFC levels progressively declined over the course of pasireotide treatment, with biochemical control (UFC < ULN) restored before completion of the 12-month core study. While it could be argued that the patient demonstrated a pattern of cyclic cortisol excess, he demonstrated progressive UFC control that was both dose related and associated with cumulative treatment.

The patient displayed progressive improvement in clinical signs and symptoms of CD over the 12-month core study. In addition, he reported improvements in QoL during the core trial and the extension phase.

The safety profile of pasireotide was consistent with that of the somatostatin analogue class, except for the degree of hyperglycemia. The most common AEs were related to transient gastrointestinal discomfort. Importantly, all AEs were manageable without reduction of pasireotide dose.

Hyperglycemia was effectively controlled by initiation of metformin treatment, which led to significant reductions in blood glucose level. Indeed, metformin dose was reduced and ultimately halted due to emergence of hypoglycemia-related AEs. Hyperglycemia associated with pasireotide treatment was reversed 1 month after discontinuation of study drug. 

These findings are in accordance with recent studies in healthy volunteers, whereby pasireotide-induced hyperglycemia was effectively managed using antihyperglycemic medication [14]. As such, the hyperglycemic response to pasireotide reported in this case study supports the monitoring of blood glucose levels of pasireotide-treated patients and prompt administration of antidiabetic medication if blood glucose levels are found to increase.

This patient's care required ongoing management of clinical symptoms, disease-related parameters, blood sugar levels, cholecystitis, and drug AEs. A low-fat, low-glycemic index diet was recommended. The patient was referred to a local dietician and to a physical therapist for a low-impact, muscle-strengthening regime. To manage side effects, ice and heat were applied locally to the injection site. Nausea was avoided by offering a protein snack prior to each injection. The patient required counselling for depression early in the course of therapy and counselling for family planning. Collaboration with the patient's primary care provider was ongoing throughout treatment. 

## 4. Conclusion

This case illustrates the successful long-term management of a patient with recurrent CD using pasireotide. Biochemical control was achieved, along with clinical and QoL improvements and effective control of AEs. This case report demonstrates a patient with a progressive response to treatment over 2 years. In addition to monitoring biochemical parameters, clinical signs and symptoms along with QoL measures represent important endpoints in the treatment of CD. Anticipation and early treatment of common drug-related side effects improve patient tolerability and promote continuity of drug use. Treatment success is a composite of all measures.
